# Different Dose-Dependent Modes of Action of C-Type Natriuretic Peptide on *Pseudomonas aeruginosa* Biofilm Formation

**DOI:** 10.3390/pathogens7020047

**Published:** 2018-04-24

**Authors:** Florie Desriac, Thomas Clamens, Thibaut Rosay, Sophie Rodrigues, Ali Tahrioui, Jérémy Enault, Lucille Roquigny, Pierre-Jean Racine, Laure Taupin, Alexis Bazire, Alain Dufour, Jérôme Leprince, Emeline Bouffartigues, Sylvie Chevalier, Marc G. J. Feuilloley, Olivier Lesouhaitier

**Affiliations:** 1 Laboratory of Microbiology Signals and Microenvironment LMSM EA 4312, Normandie Université, University Rouen-Normandy, 27000 Evreux, France; florie.desriac@plymouth.ac.uk (F.D.); thomas.clamens1@univ-rouen.fr (T.C.); thibaut.rosay@windowslive.com (T.R.); sophie.rodrigues@univ-rouen.fr (S.R.); ali.tahrioui@univ-rouen.fr (A.T.); jeremy.enault@univ-rouen.fr (J.E.); lucille.roquigny@univ-rouen.fr (L.R.); pierre-jean.racine@univ-rouen.fr (P.-J.R.); emeline.bouffartigues@univ-rouen.fr (E.B.); sylvie.chevalier@univ-rouen.fr (S.C.); marc.feuilloley@univ-rouen.fr (M.G.J.F.); 2 Laboratoire de Biotechnologie et Chimie Marines (LBCM), EA 3884, LBCM, IUEM Université de Bretagne-Sud, 56100 Lorient, France; laure.taupin@univ-ubs.fr (L.T.); alexis.bazire@univ-ubs.fr (A.B.); alain.dufour@univ-ubs.fr (A.D.); 3 Inserm U1239, PRIMACEN, Normandie Université, IRIB, Université de Rouen, 76000 Rouen, France; jerome.leprince@univ-rouen.fr

**Keywords:** biofilm, hormones, peptides, bacterial adaptation, lung, cystic fibrosis

## Abstract

We have previously shown that the C-type Natriuretic Peptide (CNP), a peptide produced by lungs, is able to impact *Pseudomonas* *aeruginosa* physiology. In the present work, the effect of CNP at different concentrations on *P. aeruginosa* biofilm formation was studied and the mechanisms of action of this human hormone on *P. aeruginosa* were deciphered. CNP was shown to inhibit dynamic biofilm formation in a dose-dependent manner without affecting the bacterial growth at any tested concentrations. The most effective concentrations were 1 and 0.1 µM. At 0.1 µM, the biofilm formation inhibition was fully dependent on the CNP sensor protein AmiC, whereas it was only partially AmiC-dependent at 1 µM, revealing the existence of a second AmiC-independent mode of action of CNP on *P. aeruginosa*. At 1 µM, CNP reduced both *P. aeruginosa* adhesion on glass and di-rhamnolipid production and also increased the bacterial membrane fluidity. The various effects of CNP at 1 µM and 0.1 µM on *P. aeruginosa* shown here should have major consequences to design drugs for biofilm treatment or prevention.

## 1. Introduction

The biofilm lifestyle largely adopted by bacteria allows their protection against environmental stresses including those induced by biocides or antibiotics [1], leading to persistency and chronic infections [2]. Indeed, biofilm-associated bacteria appear less sensitive to antibiotics than their planktonic counterparts [3]. Antibiotics have played a key role in our civilization through their ability to cure the great majority of bacterial infections, but, because of the huge diversity of bacteria, their high-speed reproduction, and adaptability, they also progressively promoted a selection of the most resistant strains. This constitutes a new challenge for treating bacterial infections [4]. In this context, the principle of a bi-therapeutic approach, in which an anti-biofilm compound is used to restore the bacterial sensitivity to co-administrated antibiotics [5,6], constitutes a promising strategy to develop new anti-bacterial treatments [7]. However, if the principle is elegant, its application is not obvious as it has been observed that bacteria can also develop resistances against anti-biofilm compounds, such as cationic antimicrobial peptides (CAMP) through activation of various sensors and transduction systems [8,9,10].

A new approach for discovering natural and potentially low toxicity antibacterial molecules consisted in studying host-bacterium communication and particularly the impact of eukaryotic signal molecules (i.e., cytokines, hormones, and neurotransmitters) on both bacterial virulence traits and biofilm formation. This idea emerged under the initiative of Mark Lyte who first demonstrated that bacteria, such as *Escherichia coli*, are sensitive to epinephrine [11]. This opened a completely new field of research known as microbial endocrinology [12]. Since then, numerous eukaryotic signal compounds able to modify bacterial physiology have been identified [13,14,15,16]. In addition to their ability to modify bacterial virulence or production of quorum sensing molecules, the impact on biofilm formation of a few of these host-produced molecules has been reported for both Gram-negative and Gram-positive bacteria. For instance, it has been shown that the neuropeptide substance P (SP) produced by skin nerves is able to enhance both *Staphylococcus aureus* and *S. epidermidis* binding to keratinocytes and favors *S. epidermidis* biofilm formation [17]. The SP spectrum of activity is large since biofilm formation by *Bacillus cereus* was also enhanced by SP [18]. The regulation of biofilm formation by neuropeptides appears complex since, in contrast to SP, the Calcitonin Gene-Related Peptide (CGRP) impairs *S. epidermidis* biofilm formation [19]. It is interesting to note that these bacterial effects of human peptides described above, are consecutive to the binding of the molecules by specific bacterial receptors/sensors acting with a similar sensitivity as their human counterparts. In addition to the effects of neuropeptides on biofilm formation, it has been reported that the small stress hormones, epinephrine, and norepinephrine, enhanced biofilm formation in *E. coli* [20,21].

Now the same question arises for *Pseudomonas aeruginosa*, for which resistance to antibiotic treatments is largely attributed to its transition from a planktonic to a biofilm lifestyle in the host [22,23,24]. The high adaptability of *P. aeruginosa* to the host environment strongly suggests that it can detect a large range of eukaryotic chemical signals [25,26]. Indeed, the neurotransmitter γ-aminobutyric acid (GABA) known to be able to bind a *Pseudomonas* protein [27], slightly inhibits both *P. aeruginosa* [28] and *P. fluorescens* biofilm formation [29]. Another study showed that the endogenous opioid dynorphin A modifies the production of *P. aeruginosa* quorum sensing autoinducers triggering bacterial physiology modifications [30]. In addition, opioid receptor agonists are able to mimic the effect of dynorphin A on *P. aeruginosa*, especially by enhancing bacterial adhesion properties [30], suggesting that this effect is relayed by a bacterial sensor. A first hypothesis suggested that dynorphin A was able to bind the global transcriptional regulator MvfR, after passive diffusion through bacterial membranes [30]. However, a recent study using new chemical probes identified the membrane sensor kinase ParS of the ParR/ParS two component system as the bacterial sensor for dynorphin A [31]. In the same vein, the cytokine Interferon-γ was shown to induce modifications in *P. aeruginosa* virulence traits via binding to the major outer membrane protein OprF [32]. OprF is a porin that fulfills multiple functions in *P. aeruginosa* [33] including regulation of virulence [34] and biofilm formation [35].

Altogether, these data show that activation of some bacterial sensors by human communication compounds favors bacterial adhesion and biofilm formation making thus these compounds not appropriate for developing therapeutic approaches. However, a much better candidate could be the C-type Natriuretic Peptide (CNP). CNP is a member of the natriuretic peptide family also including the Atrial Natriuretic Peptide (ANP) and the Brain Natriuretic Peptide (BNP). These three hormones are released by mammalian cardiomyocytes and endothelial cells in order to regulate cardiovascular homeostasis and blood pressure [36]. It has been shown that the release of natriuretic peptides (BNP and CNP) in blood is enhanced in presence of Gram-negative bacteria or the endotoxin lipopolysaccharide (LPS) [37]. In the same vein, in vitro study has demonstrated that endothelial cells exposed to LPS secreted more CNP (×25) than control cells [38]. These data suggest that during the infectious process Gram-negative bacteria, including *P*. *aeruginosa*, are exposed to natriuretic peptides secreted locally. In parallel, it has been shown that hBNP (but not hCNP) possesses an antimicrobial activity against numerous micro-organisms [39]. Altogether, these data prompted us to investigate deeper the potential activity of BNP and CNP on *P. aeruginosa*. We first observed that CNP used at 1 µM slightly enhanced *P. aeruginosa* virulence [40]. Since virulence and biofilm formation are inversely regulated in *P. aeruginosa*, we naturally investigated the effect of natriuretic peptides, and more particularly CNP, on *P. aeruginosa* biofilm formation. As expected, we observed that CNP at 0.1 µM strongly reduced the biofilm formation and that this action is mediated through specific CNP recognition by a bacterial sensor identified as the AmiC protein [41].

In the present study, we decided to investigate the dose-related effects of CNP on *P. aeruginosa* biofilm formation. Using several concentrations, we observed that CNP inhibited biofilm formation in a dose-dependent manner. The most efficient concentrations are 1 and 0.1 µM. The impact of CNP on *P. aeruginosa* at 1 µM appeared only partly relayed through binding to AmiC, suggesting that CNP has multiple modes of action on *P. aeruginosa*.

## 2. Results

### 2.1. Effect of CNP on P. aeruginosa Biofilm Formation in Dynamic Conditions

**Figure 1:**
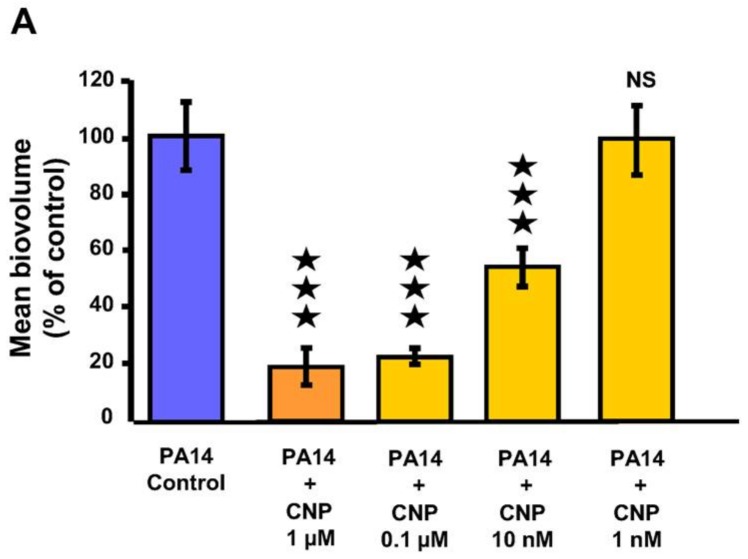

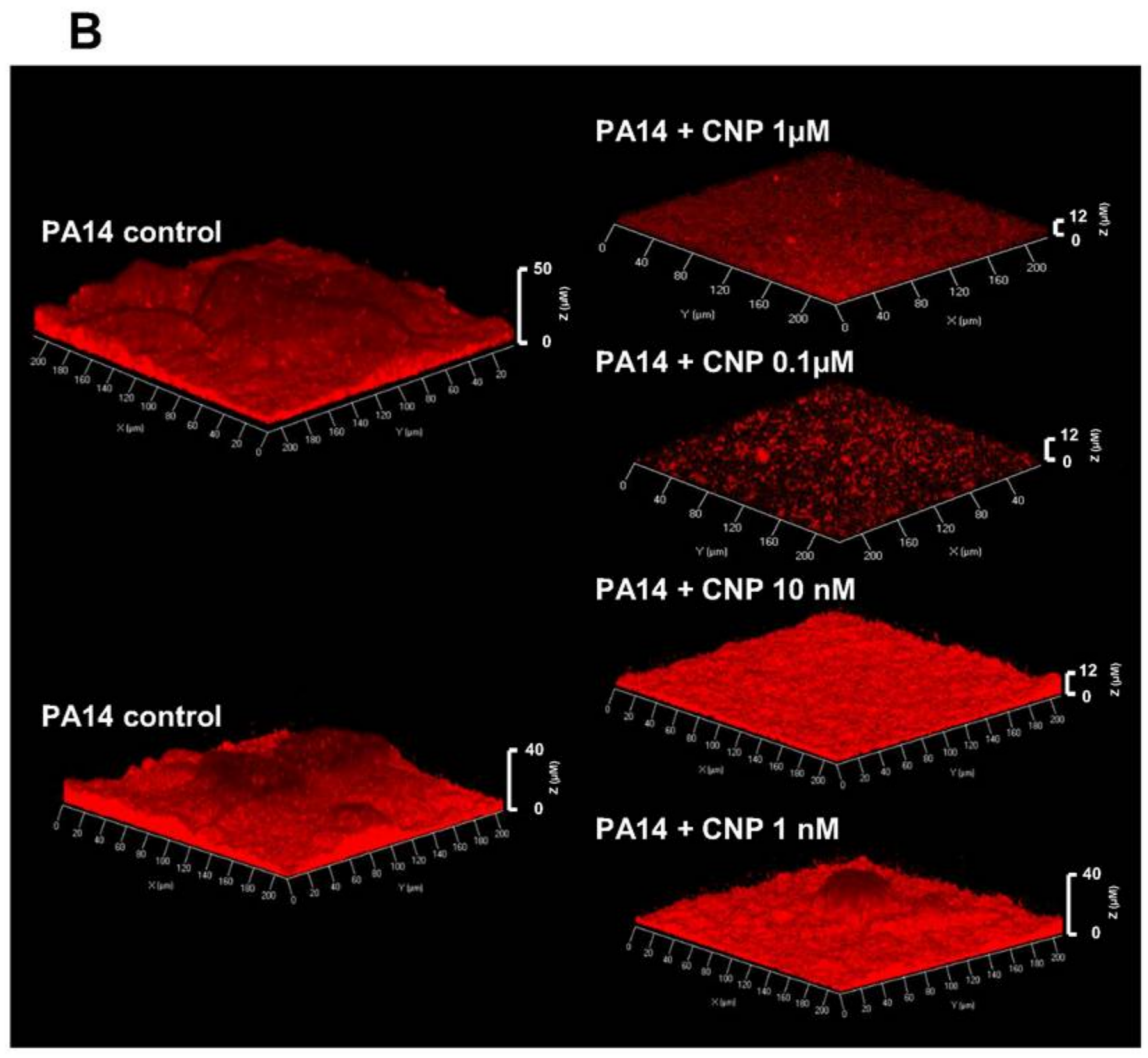
Biofilm formation in dynamic conditions by *P. aeruginosa* PA14 exposed to C-type Natriuretic Peptide (CNP). (**A**) COMSTAT analyses of biofilms of *P. aeruginosa* PA14 control (blue bar) or exposed to several concentrations of CNP (orange bars). Data are the means of four measuring fields obtained from four independent experiments. ★★★, *p* < 0.001; NS: not significantly different. (**B**) 3D-shadow representations of the biofilm structures developed under dynamic conditions by *P. aeruginosa* PA14 control (left images) or exposed to CNP at 1 µM, 0.1 µM, 10 nM or 1 nM (right images). Biofilms were stained with the Syto 61 Red dye and observed by confocal laser scanning microscopy.

**Figure 2:**
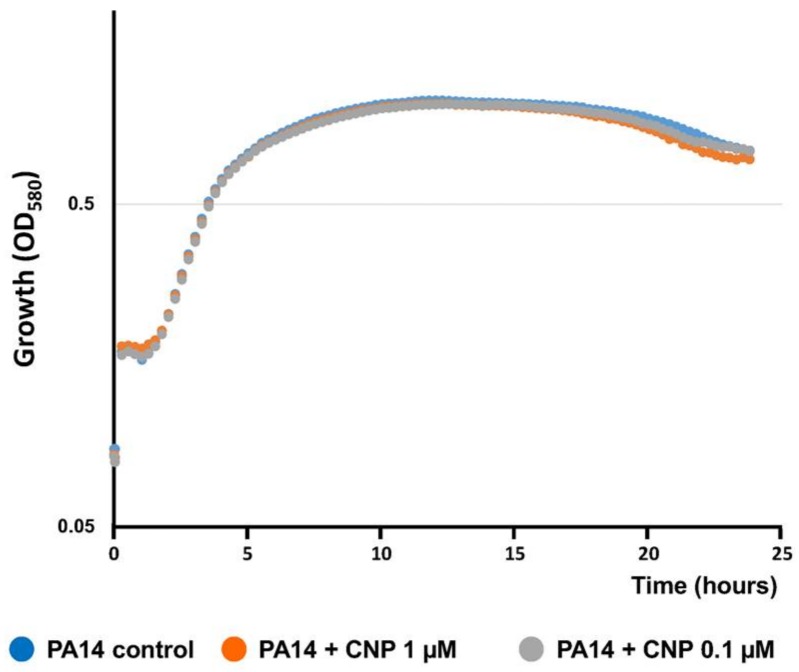
Growth of PA14 exposed to C-type Natriuretic Peptide (CNP). Culture growth curves were measured at OD_580nm_. Absorbance was recorded every 15 min for 24 h. Curves are color-coded as follow: PA14 WT alone (blue), PA14 WT exposed to CNP (1 µM) (orange) and PA14 WT exposed to CNP (0.1 µM). Results are the mean of eight replicates from three independent experiments. The same kinetics have been observed when PA14 WT was exposed to CNP (10 nM and 1 nM).

We previously observed that CNP at a concentration of 0.1 µM strongly inhibited (−78%; *p* < 0.001) the formation of *P. aeruginosa* PA14 biofilms grown for 24 h on glass slide under a constant medium flow [41]. Administration of CNP at 1 µM in the same condition induced a strong (−81.0 ± 6.3%) and significant (*p* < 0.001) decrease of the biofilm production when compared to biofilms grown without CNP (Figure 1A). In addition, we observed that CNP used at 10 nM for 24 h reduced by 46.9 ± 6.7% (*p* < 0.001) the *P. aeruginosa* ability to form a biofilm (Figure 1A). In parallel, we observed that at this concentration the CNP reduced the built-up of mushroom-like structures (Figure 1B). Finally, CNP used at 1 nM failed to affect *P. aeruginosa* biofilm formation (Figure 1A,B). Taken together, these results show that CNP impedes biofilm development of *P. aeruginosa* in a dose-dependent manner when tested in dynamic conditions. In order to evaluate any CNP impact on *P. aeruginosa* growth, we studied the impact of several CNP concentrations on the growth kinetisc of *P. aeruginosa* PA14 in liquid medium. None of the tested CNP concentrations (i.e., 1 µM, 0.1 µM, 10 nM and 1 nM) affected bacterial growth (Figure 2). Therefore, the defect in biofilm development observed on Figure 1A in the presence of 10 nM to 1 µM CNP cannot be explained by lower growth rates.

### 2.2. Involvement of AmiC in the CNP Effect on P. aeruginosa

**Figure 3:**
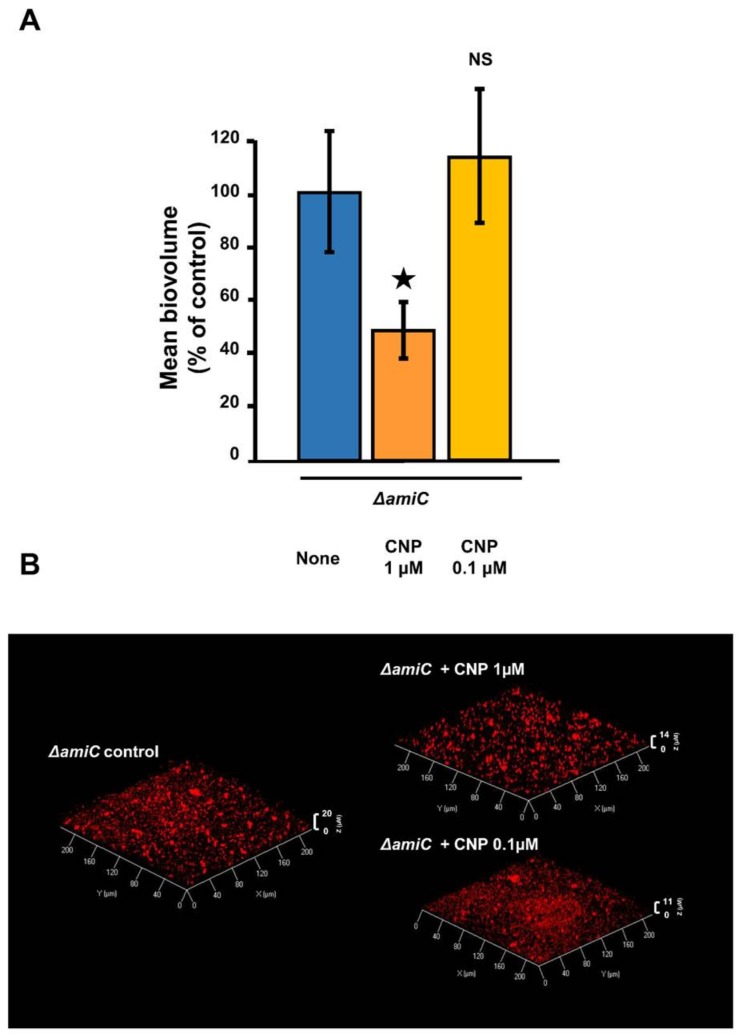
Biofilm formation in dynamic condition by *P. aeruginosa* PA14 mutant strain Δ*amiC* exposed to C-type Natriuretic Peptide (CNP). (**A**) COMSTAT analyses of biofilms of Δ*amiC* mutant strain (blue bar) exposed to CNP (1 µM; deep orange bar) or to CNP (0.1 µM; light orange bar). Data are the means of twenty-one samples from seven independent experiments for Δ*amiC* control condition, six samples from two independent experiments for Δ*amiC* exposed to CNP (1 µM) and twelve samples from four independent experiments for Δ*amiC* exposed to CNP (0.1 µM). ★, *p* < 0.05; NS: not significantly different. (**B**) 3D-shadow representations of the biofilm structures developed under dynamic conditions by *P. aeruginosa* Δ*amiC* control (left part) or exposed to CNP at 1 µM or 0.1 µM (right part). Biofilms were stained with the Syto 61 Red dye and observed by confocal laser scanning microscopy.

We previously demonstrated that the effect of 0.1 µM CNP on biofilm formation requires the binding of CNP to the bacterial sensor AmiC [41]. We first observed that the ∆*amiC* mutant strain ability to form a biofilm is strongly impaired (Figure 3B). When the ∆*amiC* mutant strain was exposed to 0.1 µM CNP, the biofilm formation was not affected as compared with the corresponding control (Figure 3A,B). In contrast, when the CNP concentration was increased to 1 µM, the ∆*amiC* strain remained partially sensitive to the peptide since the biofilm was reduced by 51.4 ± 10.4% (*p* < 0.05) compared to biofilm of the same mutant grown without CNP (Figure 3A,B).

### 2.3. Effect of CNP on P. aeruginosa Adhesion Properties

**Figure 4:**
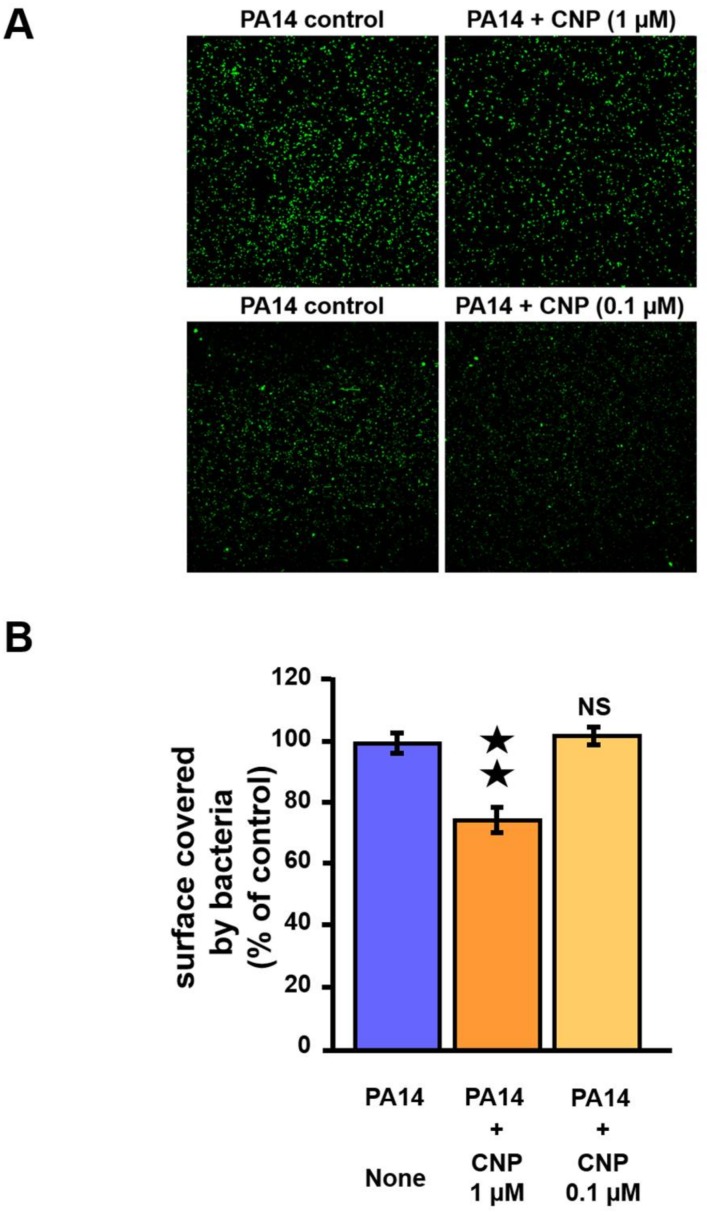
Adhesion on glass slide of *P. aeruginosa* PA14 exposed to C-type Natriuretic Peptide (CNP). (**A**) Pictures representing the occupancy of bacteria control (left part) or bacteria exposed to CNP (1 µM) or to CNP (0.1 µM) (right part) on glass slides. Bacteria were stained with Syto9 green dye. (**B**) Percentage of recovery using ImageJ [43]. The data are the mean of six measurements realized from two independent experiments. ★★, *p* < 0.01. NS: not significantly different.

The formation of biofilms depends on the motility of a bacterium to colonize its environment as well as its ability to attach to biotic and abiotic surfaces [42]. We observed that exposition to 1 µM of CNP led to reduce the ability of the bacteria to attach to glass slides under static condition (Figure 4A). More precisely, the surface occupied by bacteria after exposure to CNP is reduced by 24.8% (*p* < 0.01) compared to control condition (i.e., *P. aeruginosa* not exposed to CNP peptide) (Figure 4B). In contrast, exposition to 0.1 µM of CNP did not modify the ability of the bacteria to attach to glass slides under static condition (Figure 4A,B).

### 2.4. Effect of CNP on P. aeruginosa Di-Rhamnolipid Production

**Figure 5:**
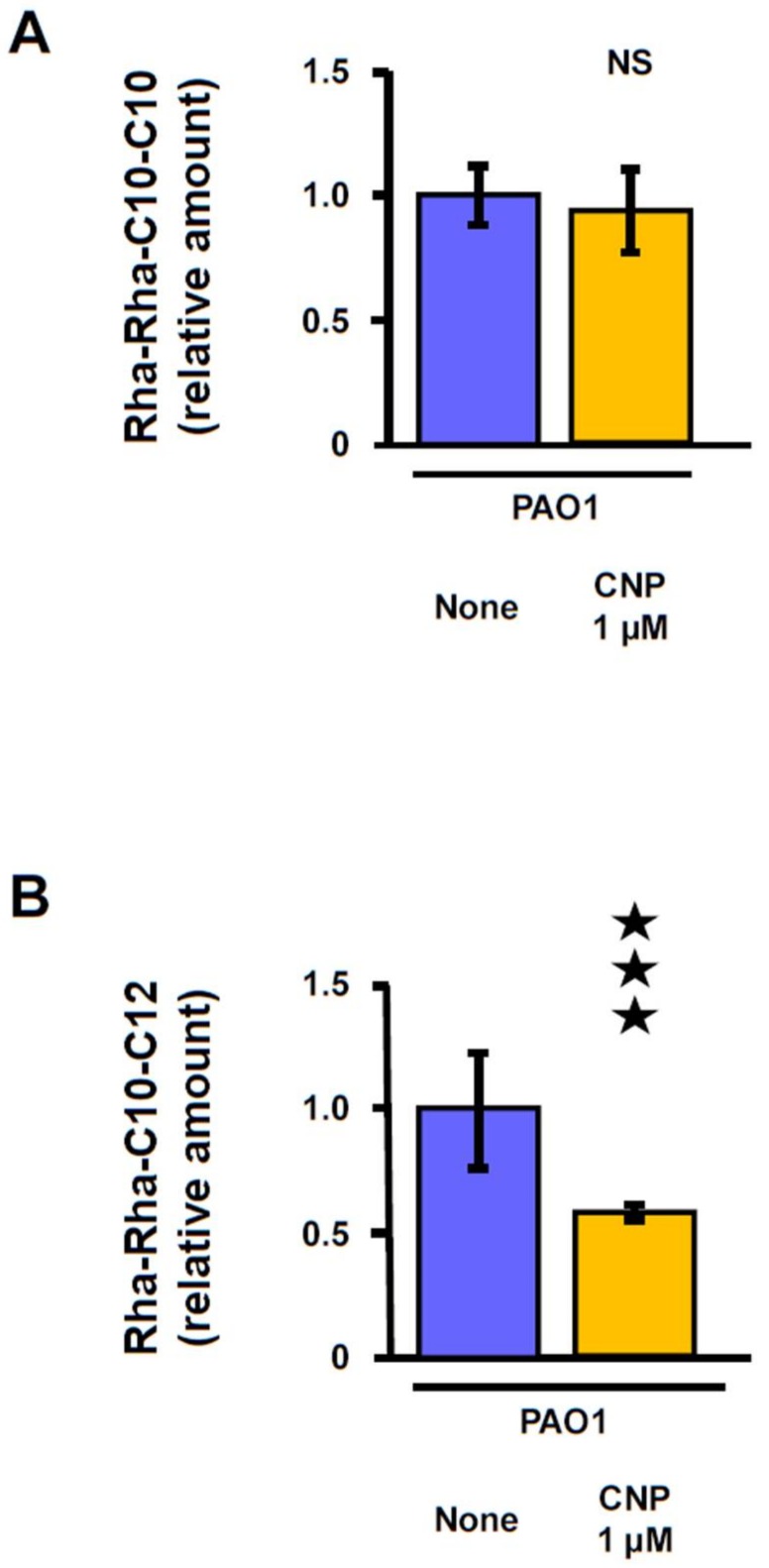
Effect of C-type Natriuretic Peptide (CNP) on rhamnolipids production by *P. aeruginosa* PAO1. Relative amounts of the di-rhamnolipid Rha-Rha-C10-C10 (**A**) and the di-rhamnolipid Rha-Rha-C10-C12 (**B**) in *P. aeruginosa* PAO1 supernatants, 4 h later bacteria exposure to physiologic water (control, blue bars) or CNP 1 µM (orange bars). Data are the means of three independent experiments. The mean of [Rha-Rha-C10-C10] and [Rha-Rha-C10-C12] levels in control conditions were 174 × 10^3^ Aera/OD_580_ and 9.7 × 10^3^ Aera/OD_580_, respectively. NS: not significantly different. ★★★, *p* < 0.001.

Rhamnolipids are secreted biosurfactants composed of mono- or di-rhamnose linked to a fatty acid chain of variable length [44]. These glycolipids are surface-active and affect biofilm formation through microcolonies formation, surface motility [45]. They also contribute to the biofilm architecture by maintaining fluid channels between mushroom-like structures [46]. In order to evaluate the impact of CNP on *P. aeruginosa* rhamnolipid production, we quantified the two most abundant di-rhamnolipid species produced by the PAO1 strain: Rha-Rha-C10-C10 and Rha-Rha-C10-C12 [47]. We switched to the PAO1 strain for this experiment because of our background on rhamnolipid production by this strain [39] and because we have previously observed that PAO1 strain and PA14 strain show the same sensitivity to CNP (1 µM) [41]. We observed that CNP (1 µM) did not modify the quantity of Rha-Rha-C10-C10 produced after 6 h of culture (Figure 5A). In contrast, we noted that CNP induced a deep fall (−41.1 ± 3.0%; *p* < 0.001) in the amount of Rha-Rha-C10-C12 produced by PAO1 after 6 h of culture (Figure 5B).

### 2.5. Effect of CNP on P. aeruginosa Membrane Fluidity

**Figure 6:**
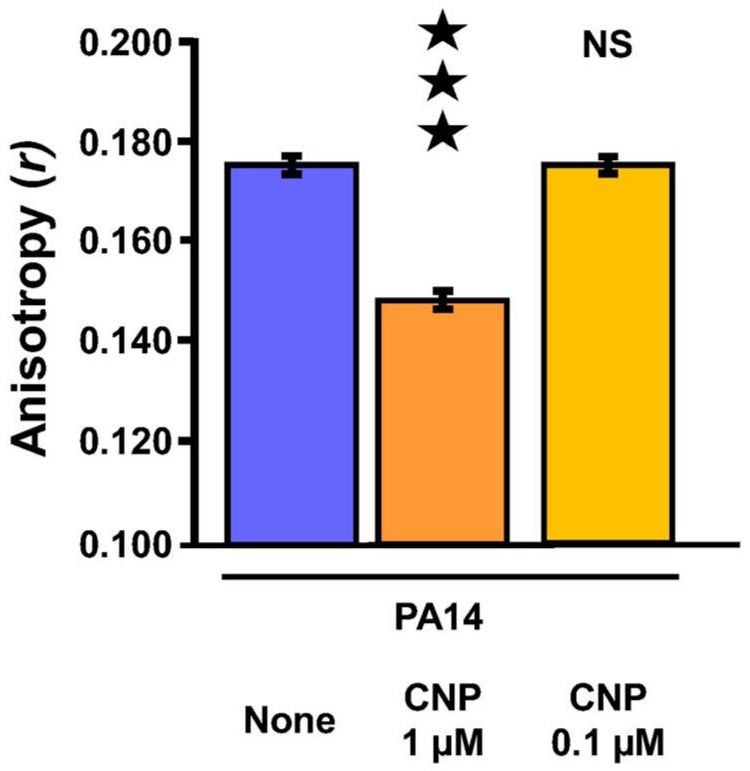
Membrane fluidity of *P. aeruginosa* PA14 exposed to C-type Natriuretic Peptide (CNP). The fluorescence anisotropy *r*-values for PA14 strain used as control (blue bar), PA14 exposed to CNP at 1 µM (deep orange bar) and 0.1 µM (light orange bar) were obtained from 100 different wells from five independent experiments for PA14 control condition (blue bar), in 76 different wells from four independent experiments for PA14 exposed to CNP (1 µM; deep orange bar) and in 32 different wells from two independent experiments for PA14 exposed to CNP (0.1 µM; light orange bar). An inverse relationship occurred between the fluorescence anisotropy and cell membrane fluidity. ★★★, *p* < 0.001; NS, not significantly different.

Since attachment to surfaces and secretion of biofilm matrix molecules are both depending on bacterial membrane fluidity [48] and since we observed a contrast concerning the mode of action of CNP on *P. aeruginosa* when the peptide was utilized at 1 µM and 0.1 µM, we evaluated the CNP impact on *P. aeruginosa* membrane fluidity by measuring the fluorescence polarization anisotropy (*r*) of 1,6-diphenyl-1,3,5-hexatriene (DPH), a lipophilic fluorescent probe. An inverse relationship occurs between measured DPH fluorescence anisotropy and membrane fluidity [49]. Statistical analysis of *r*-values shows that exposure of *P. aeruginosa* PA14 strain to 1 µM of CNP for 3 h triggered an enhancement of the membrane fluidity (*p* < 0.001) (Figure 6). By contrast, exposure of *P. aeruginosa* PA14 strain to 0.1 µM of CNP for 3 h failed to affect the membrane fluidity (Figure 6). The CNP influence on membrane fluidity may possibly explain a part of its effect on *P. aeruginosa* biofilm formation at 1 µM.

## 3. Discussion

**Figure 7:**
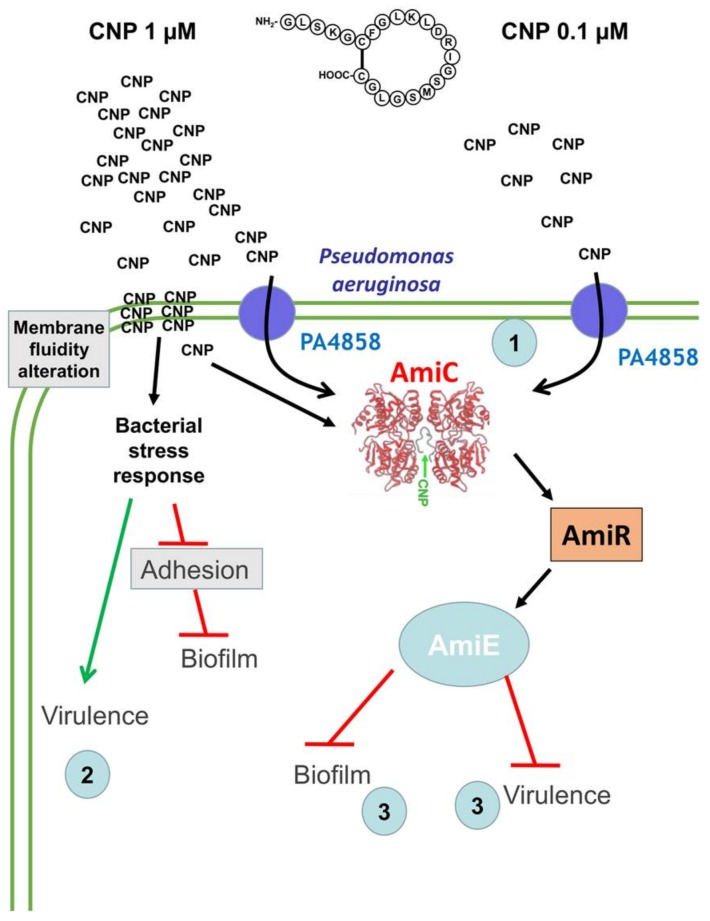
Schematic model representing the mechanism of action of the CNP on *P. aeruginosa* depending on the concentration in the vicinity of the bacteria. CNP could bind AmiC sensor after directly entering into the bacteria (CNP 1 µM) or through PA4858 [41] (1) (CNP 1 µM and 0.1 µM). The direct entrance of CNP (1 µM) could trigger bacterial stress response explaining the enhancement of virulence previously described [40] (2), and the reduction of both adhesion and biofilm formation. In parallel, the binding of CNP to AmiC will release AmiR protein that allows transcription of the whole *ami* operon leading to an overproduction of the AmiE enzyme that was shown to reduce both biofilm formation (3) and virulence (3) [52].

We have previously shown that CNP, which is produced by lung cells [50], is able to modify *P. aeruginosa* physiology. More precisely, we observed that *P. aeruginosa* cytotoxicity [51] and virulence [40] are increased after CNP exposure, while biofilm formation is strongly inhibited [41]. In the present study, we observed that CNP has similar efficiencies when used at 0.1 and 1 µM (biofilms reduced by about 80%), and that a concentration of 10 nM is still able to inhibit by half the capacity of *P. aeruginosa* to form a biofilm in dynamic conditions. CNP thus inhibits *P. aeruginosa* biofilm formation in a dose-dependent manner in the 10 nM–0.1 µM range of concentrations. Furthermore, our data indicate that its mechanism of action depends on the peptide concentration in the 0.1–1 µM range: while the impact of 0.1 µM CNP is fully relayed by the AmiC sensor, the CNP anti-biofilm effect observed at 1 µM seems to be only partly relayed by AmiC. Taken together, these data suggest that CNP uses another not-yet elucidated pathway to achieve its full effect on the bacteria (Figure 7).

The next step of our study was dedicated to the identification of the putative additional effect of CNP when the peptide is used at 1 µM. It was shown that another member of the natriuretic peptide family, the Brain Natriuretic Peptide (BNP) exhibited an antimicrobial activity against a large spectrum of micro-organisms [39]. This effect was observed only at high peptide concentration (36 µM). Since we have previously observed that 1 µM BNP mimicked fully the CNP effect on *P. aeruginosa* cytotoxicity traits [51], and partly on production of virulence factors [40], and inhibited biofilm formation with the same efficiency as CNP [41], we investigated a possible anti-bacterial effect of the CNP peptide. We ruled out this hypothesis since CNP has no impact on *P. aeruginosa* growth at any tested concentration. It was shown that several natriuretic peptides including CNP and CNP-derived peptide were able to affect membrane structures by forming pores in reconstituted membrane [53,54], suggesting that CNP could insert himself in biological membranes, similarly to CAMP peptides such as magainin-2 or defensins [55]. In this context, we investigated the possible influence of CNP on membrane fluidity. Using a fluorescent probe, we observed that CNP used at 1 µM enhances significantly *P. aeruginosa* membrane fluidity whereas at the concentration of 0.1 µM the peptide has no influence on membrane fluidity. These data strongly suggest that the part of the effect provoked by CNP at 1 µM on *P. aeruginosa* biofilm formation that was not supported by AmiC binding could be due to a non-specific effect involving a membrane fluidity alteration.

Another possible explanation is that CNP could modify the matrix production during biofilm formation or could affect the bacterial ability to attach onto glass slides. In agreement with this hypothesis, we observed that *P. aeruginosa* exposed to CNP at 1 µM produced two-fold less di-rhamnolipid Rha-Rha C10-C12. This effect appeared highly specific to CNP since BNP, used at the same concentration, had no impact on rhamnolipid production (data not shown). In addition, using qRT-PCR approach we have observed that the expression level of *envC*, which was described to be involved in intrinsic resistance to CAMPs [56], is reduced by 40% after treatment with CNP (1 µM; 3 h) (data not shown). The resistance mediated by EnvC has been previously linked to increased surface negativity and hydrophobicity [56]. The higher hydrophobicity generated by the lower expression of EnvC in response to CNP may thus explain a less efficient adhesion of CNP-treated cells onto hydrophilic borosilicate glass used in dynamic condition [57].

To study adhesion, the first step of biofilm construction, the choice of the technique used (i.e., dynamic or static) is important [58]. In this case, the continuous flow approach allows to explore the importance of adhesion in the really early step of biofilm construction [58], knowing that the first 20 min are determinant for the success of this process [59]. We observed in the present study that CNP used at 1 µM significantly reduced the glass surface covered by *P. aeruginosa* after 2 h. This was supported by the fact that the *pilY1* mRNA level is two-fold decreased 3 h after the addition of CNP (1 µM) (data not shown). The PilY1 protein of *P. aeruginosa* has been shown to be essential for type IV pilus assembly and evidently contributes to cell adhesion and virulence [60,61]. Even if these expression data were obtained from planktonic culture, CNP could be able to repress pilus formation, which can explain the observed decrease in attachment to glass.

Another hypothesis is the possible trigger of bacterial stress induced by CNP at 1 µM that finally could explain the default in biofilm formation through bacterial membrane fluidity enhancement [48]. Indeed, it has been proposed that *P. aeruginosa* cells within biofilms reduced their membrane fluidity through an enhancement of the number of saturated fatty acids into the membrane [48]. Herein, we observed that CNP both enhances membrane fluidity and reduces biofilm formation. We can speculate that the impact of CNP on membrane fluidity could reduce the stability of the biofilm during its formation process. It has been shown that the extra cytoplasmic function sigma factor SigX is involved in membrane fluidity modification through fatty acids synthesis regulation [62]. This phenomenon allows *P. aeruginosa* to quickly adapt its physiology during stresses. In this context, we observed by qRT_PCR assays that when *P. aeruginosa* is exposed to 1 µM of CNP the level of *sigX* mRNA is increased by 70%, 3 h after the addition of CNP (data not shown).

Besides these data obtained in planktonic condition, we cannot neglect the role played in the biofilm formation by the *ami* operon products and especially the enzyme amidase AmiE, since this operon is over-expressed when the bacteria are exposed to CNP [41]. Indeed, the *amiE* mRNA production was demonstrated to be increased 18 and 34 fold when bacteria were grown in biofilm compared to planktonic and dispersed states, respectively [63]. In addition, the AmiE protein concentration was shown to be regulated during biofilm formation since its amount was strongly enhanced when compared with bacteria grown in planktonic state [64,65]. The crucial role of AmiE in the CNP-related effect on biofilm formation was finally confirmed by a study showing that a *P. aeruginosa* strain over-producing the AmiE enzyme was strongly impaired in its ability to form a biofilm [52] in conditions identical to the one used in the present study. 

In conclusion, our data show that the contact between the CNP at 1 µM and *P. aeruginosa* triggers a bacterial response that finally decreases the biofilm formation ability and activates a stress answer favoring bacterial virulence (Figure 7). This dual effect is abolished when the peptide is less concentrated (0.1 µM), which induces only a strong reduction of biofilm formation after CNP binding to AmiC. Taken together, these data highlight an interesting future use of CNP as a potential anti-*P. aeruginosa* biofilm agent, and strongly suggest that 0.1 µM concentration would be a suitable window for a therapeutic approach. However, it must be kept in mind that hormones in general, and CNP especially, are present at low concentration in healthy human blood. Since natriuretic peptides are already used as drugs in humans [66,67] and since CNP exerts both anti-inflammatory and anti-fibrotic activities in mouse lungs [68], side effects of CNP used at 0.1 µM cannot be excluded and should be evaluated in pre-clinical trials. Nevertheless, whatever the outcome of this essential evaluation in pre-clinical trials, our study has allowed for identification of bacterial AmiC as the sensor activated by CNP. Identifying this kind of bacterial target is a good start to design new drugs able to bind and activate the “hormonal” bacterial sensor without affinity for the natural receptor expressed in human cells. Taken together, our work reinforces the idea that studying the impact of hormones on bacteria virulence will allow to identify new targets in bacteria to counteract their pathogenicity.

## 4. Materials and Methods

### 4.1. Bacterial Cultures and Tested Molecules

*Pseudomonas aeruginosa* PA14 wild-type and PA14 Δ*amiC* strains were obtained from Harvard Medical School (Boston, MA, USA) [69] and kindly provided by the Biomerit Research Centre (Univ. Cork, Cork, Ireland). *P. aeruginosa* PAO1 strain was obtained from an international collection [40]. Bacterial cultures realized in Luria Bertani (LB) medium were exposed to the tested molecule—the human CNP (CNP1-22 (Gly-Leu-Ser-Lys-Gly-Cys-Phe-Gly-Leu-Lys-Leu-Asp-Arg-Ile-Gly-Ser-Met-Ser-Gly-Leu-Gly-Cys [disulfide bridge: 6–22]); Polypeptide, Strasbourg, France), resuspended in sterile water or to sterile water alone (control). The density of the bacterial suspension was determined by absorption at 580 nm using a spectrophotometer (ThermoSpectronics, Cambridge, UK). The bacterial density and the absence of contamination were controlled by plating.

### 4.2. Kinetics of Bacterial Growth

For the monitoring of bacterial growth, an overnight bacterial culture in LB medium was diluted at OD_580_ = 0.08 in fresh LB medium. Bacterial culture was dispatched (2 mL for each condition) with CNP diluted at different concentrations (1 µM to 1 nM) or without CNP (control condition). Immediately after, 200 µL of each bacterial suspension were dropped in a sterile 100-well flat-bottomed plastic culture plate (Honeycomb, Bioscreen, Helsinki, Finland). The plate was incubated for 24 h at 37 °C under constant agitation in a Bioscreen C. The OD of each well was measured at 580 nm every 15 min.

### 4.3. Biofilms Formation on Glass Slides Under Dynamic Conditions and Adhesion Analysis

Biofilms formation and adhesion were performed under hydrodynamic conditions in a three-channel flow cell (1 mm × 4 mm × 40 mm Biocentrum, DTU, Denmark) [70]. The flow system was assembled, prepared and sterilized as described by Tolker-Nielsen and Sternberg [71]. The substratum consisted of a microscope glass coverslip (24 × 50 KnittelGlasser, Braunschweig, Germany). Bacteria were grown at 37 °C in 10 mL LB broth under shaking. After 2 h, CNP was added (1 µM to 1 nM) and bacterial growth was pursued for 3 h. Bacterial cells were then harvested by centrifugation (10 min, 7500 rpm) and washed twice with sterile physiological solution (NaCl 0.9%).

Each channel of the flow cell was inoculated with 300 µL of bacterial suspension diluted to an OD_580nm_ of 0.1. A 2 h attachment step was performed without any flow. LB medium with or without CNP (1 µM to 1 nM) was then pumped with a 2.5 mL·h^−1^ flow during 24 h.

Adhered cells or biofilms formed by *P. aeruginosa* were stained with fluorescent dyes and observed by confocal laser scanning microscopy (CLSM). Cells were stained by injecting into each flow cell channel 300 µL of 5 µM Syto61 or Syto9 (Molecular Probes, Invitrogen, Carlsbad, CA, USA) prepared in sterile physiological solution (0.9% NaCl, SPS), incubated at room temperature for 15 min in the dark and then washed for 15 min by a flow of medium (2.5 mL·h^−1^). CLSM observations were then immediately performed with a Zeiss LSM710 (Carl Zeiss Microscopy, Oberkochen, Germany) using a 40× oil immersion objective. Syto 9 and Syto 61 were excited at 488 and 633 nm respectively. Fluorescence emission was detected from 500 to 550 nm for Syto 9 and from 640 to 660 nm for Syto 61. Images were taken every micrometer throughout the whole biofilm depth. For visualization and processing of three-dimensional (3D) image, the Zen 2.1 SP1 software (Carl Zeiss Microscopy, Oberkochen, Germany) was used. Quantitative analyses of images stacks were performed using the COMSTAT software (http://www.imageanalysis.dk/) [72]. For attachment assays, the percentage of surface covered by bacteria was evaluated using ImageJ software (National Institutes of Health, Bethesda, MD, USA).

### 4.4. Fluorescence Anisotropy Microplate Assays

Fluorescence anisotropy analysis of PA14 was performed as described by Vincent et al. [49] with a few modifications. Bacteria were grown at 37 °C in 10 mL LB broth under agitation; after 2 h growth CNP was added (1 µM or 0.1 µM) and bacterial growth was pursued for 3 h. Bacterial cells were then harvested by centrifugation, washed twice in 10 mM MgSO_4_ (5 min, 7500 rpm) and resuspended in the same wash solution to an OD_580_ of 0.1. 1 µL of a 4 mM stock solution 1,6-diphenyl-1,3,5-hexatriene (DPH) (Sigma-Aldrich, Saint-Quentin Fallavier, France) in tetrahydrofuran was added to a 1 mL aliquot of the resuspended cultures and incubated in the dark for 30 min at 37 °C to allow the probe to incorporate into the membrane bilayer. Measurement of the fluorescence polarization was performed using the Spark 20M multimode Microplate Reader, a polarization spectrofluorimeter from Tecan Group Ltd., Männedorf, Switzerland equipped with Te-Cool^TM^, an active temperature regulation system. Excitation and emission wavelengths were set to 365 and 425 nm, respectively, and the anisotropy *(r)* was calculated according to Lakowicz [73]. Three measurements were performed for each sample and data were recorded using SparkControl^TM^ software (Version 2.1, Tecan Group Ltd., Männedorf, Switzerland). Fluorescence polarization and membrane fluidity are inversely related since increasing anisotropy values correspond to a less fluid membrane environment and vice versa. All values are reported as means of triplicate analyses for each experimental variable.

### 4.5. Rhamnolipid Quantification

Rhamnolipids were extracted and characterized as described previously [74,75]. *P. aeruginosa* PAO1 was grown in nutrient Broth n°2 medium (BNO) (AES, Bruz, France) with either physiologic water (control) or CNP at 37 °C, in shaking conditions at 180 rpm. After 6 h of incubation, bacterial cells were collected and centrifuged at 7000 *g* for 10 min. The supernatant was filtered (0.22 µm) and analyzed by reverse-phase liquid chromatography coupled with negative-ion electrospray ionization and ion trap mass spectrometry (LC-MS-MS). For each analysis, 20 µL were injected onto a C18 HPLC column (200 × 2 mm, particle size 5 µm). A water-acetonitrile gradient containing 4 mM of ammonium acetate was used. During 4 min, the percentage of acetonitrile in water was kept at 35%, increased to 50% during 5 min, remained at 50% for 6 min and raised to 90% during last 15 min. The HPLC flow rate was 200 µL/min but only 20% was introduced into the mass spectrometer. The electrospray source parameters were: pressure nebulizer, 15 psi; dry gas flow, 7 L/min, dry temperature, 300 °C. Mass scan was extended from 50 to 800 *m/z*. The proportion of each rhamnolipid was quantified from the corresponding *m/z* [M − H]-chromatograms by the measure of peak areas.

## 5. Statistical Analysis

The non-parametric Mann-Whitney test was used to compare the means within the same set of experiments using the Past3x software.
